# Research on cross-cultural adaptation and educational management of international students in China: Case of African students at Zhejiang Normal University

**DOI:** 10.3389/fpsyg.2022.1009658

**Published:** 2022-10-17

**Authors:** Weifeng Di, Sijia Zhang, Xing Lian, Mohamed Oubibi, Dana Li, Lisi Ding, Zhujia Zhang, Tianping Yang

**Affiliations:** ^1^College of Teacher Education, Zhejiang Normal University, Jinhua, China; ^2^Key Laboratory of Intelligent Education Technology and Application of Zhejiang Province, Zhejiang Normal University, Jinhua, China

**Keywords:** African students studying in China, education management, community with a shared future for mankind, cross-cultural adaptation, international student

## Abstract

With the closer China-Africa relations and the vigorous development of the country’s education for international students, the number of African students studying in China has surged, and the difficulty of education management in colleges and universities has increased accordingly. Many scholars have conducted in-depth research on the educational management model, educational management policy, educational management status and optimization strategy for international students in China. However, the existing research dimensions are relatively single and lack of comprehensiveness. This study takes the educational management practice of African international students in Zhejiang Normal University as the breakthrough point and introduces the investigation of international students’ cross-cultural adaptability. A sample of African students coming to China is selected from Zhejiang Normal University (*n* = 475). This study designs a questionnaire based on the dimensions of enrollment management, teaching management, and daily management combined with the teaching satisfaction, cultural adaptability, and emotional attitude of African international students in China. After data analysis with SPSS 26 and AMOS 24, it can be found that enrollment management is significantly positively correlated with the time in China. Teaching management is positively correlated with age, educational background and it is positively correlated with enrollment management. Differentiated management is positively correlated with Chinese proficiency, and it is positively correlated with enrollment management and teaching management. The recognition of management staffs is positively correlated with enrollment management, teaching management and differentiated management. Teaching satisfaction is positively correlated with enrollment management, teaching management, differentiation management and recognition of management staffs. Cultural adaptation is significantly positively correlated with Chinese proficiency, enrollment management, teaching management, differentiated management, recognition of management staffs, and teaching satisfaction. Emotions and attitudes are significantly positively correlated with age and time in China, and it is positively correlated with teaching management, the recognition of management staffs, teaching satisfaction, cultural adaptation, and academic qualifications. Through in-depth analysis, it can be found that there exist problems in the current education management of African international students, such as insufficient enrollment channels, incomplete enrollment promotion, unreasonable curriculum settings, inadequate implementation of the tutorial system, and excessive differentiated management. In order to further improve the quality of educational management for African international students in China, countermeasures such as adhering to the idea of “a community with a shared future for mankind” to optimize the enrollment management; advocating the value of “harmony but different” to improve teaching management; implementing the “people-oriented” service tenet to improve daily management; deepening community value identification to promote students’ cross-cultural adaptation should be implemented.

## Introduction

Along with the trend of economic globalization, countries in the global village are more closely connected (Lule, 2021). The proposal of “a community with a shared future for mankind” is a great historical progress. It promotes development in various fields, including high quality and international development of education (Xiao and Zhong, 2020). As a community with a shared future that shares weal and woe, China and Africa regard youth as a new force in the historical process of promoting China and Africa to achieve national independence and explore the path of modernization (Steger, 2017). At the same time, promoting the development of China-Africa traditional friendship through youth exchanges has always been an important issue in China-Africa relations (Liang and Zhang, 2019; Zhao, 2021). With the joint efforts of China and Africa, African students who come to China for exchange and study have become the most representative group to fulfill the mission of China-Africa youth exchange (Liang and Zhang, 2019; Zhao, 2021). In the background of the continuous deepening of China-Africa educational cooperation, the number of African students studying in China has exceeded 80,000. The surge of African students studying in China promotes the healthy development of China-Africa relations and lays a foundation for enhancing China’s cultural soft power and implementing the Belt and Road policy. At the same time, the scale and quality of international students have become important indicators to measure the level of international development of higher education in a country. And as an important driving factor for the development of national education, the education of international students in colleges and universities and the satisfaction of international students with the educational services have received attention from all sides. However, the huge scale of African students studying in China has also brought unprecedented challenges to university education administrators (Zhao, 2021). Currently, one of the difficulties faced by Chinese higher education is how to ensure the implementation of good educational management while receiving a large number of African students in China. Although more and more scholars have paid attention to issues related to the education of international students in China and carried out in-depth research on the educational management model, educational management policy, educational management status and optimization strategy for international students in China (Liang and Zhang, 2019; Zhao, 2021), there are still some deficiencies in the education management of international students. For example, since most of the researchers are front-line practitioners, most of the research they conduct only focuses on one aspect of the management of international students in China, which lacks comprehensiveness (Usunier, 1998; Geng et al., 2020). Accordingly, the conclusions drawn from these studies are unlikely to generalize in practice. In addition, there is a lack of research achievements on the educational management of African students studying in China, which is asymmetric with the number of African students studying in China increased year by year (Gbollie and Gong, 2019; Niu et al., 2021). Besides, part of the research on the education management of African international students in China are conducted in the context of the “Belt and Road Initiative,” but in terms of scale and achievements, it is far inferior to the research on the education management of international students in China, lacking systematises and integrity, and relevant research needs to be carried out urgently (James-MacEachern and Yun, 2017; Gbollie and Gong, 2019).

Therefore, this study selects Zhejiang Normal University, a typical school for African study which has a large group of African students represented various majors at all academic credentials as the research object, conducts questionnaires and interviews with African students in China in respect of enrollment management, teaching management, and life management that involves the university’s enrollment policy and logistical support for African students compared to other studies. Data, find out the problems of African students in China in these aspects and analyze the causes by exploring their influences on three dimensions (teaching satisfaction, cultural adaptation, emotions and attitudes), and build an educational management mechanism for African students in China from the perspective of enhancing mutual understanding and cooperation between China and African and the prospect of a community with a shared future for mankind to enrich the research results in this field and provide colleges and universities for African students. China International Students Education Management provides some constructive comments.

## Literature review

### Research on the management model of international students in China

As Lozano and other scholars have found, for higher education institutions to continue developing, they must achieve innovative changes in management models (Lozano et al., 2013). At present, the management mode of international students in China has changed from the “centralized management” stage to the “decentralized management” stage and then gradually moved towards the “convergence” management mode. Convergence development in higher education is a process, not a single endpoint (Becher and Trowler, 2001). Convergent management requires roughly the same management methods for international students and domestic students (Alajlan, 2016). Wen and Tian (2022) explored the convergent management system of Chinese and international students at Tsinghua University, including admission education, foreign affairs management, and Chinese-foreign integration (Wen and Tian, 2022). Jiang (2021) focused on the current situation of convergence management of international students from countries along the Belt and Road and pointed out that cultural background diversity, low admission standards, and insufficient teaching soft power are negative factors that affect convergence management (Jiang, 2021). Liu and Liu (2021) compared the institutional systems of international student management in China and Canada, arguing that Canada’s international education system is characterized by a hands-off approach that treats international students with no or limited discrimination (Liu and Liu, 2021). China’s education system is characterized by a hand-held approach, focusing on active management, and treating international students more as a separate group. Dobbins and Knill (2009) examine the convergence process of higher education in Eastern and Central Europe from the perspectives of policy convergence, policy legacy and path dependence (Dobbins and Knill, 2009). Gatfield et al. (1999) found that taking into account the characteristics of ethnic groups, establishing a unified standard of academic guidance is the key to determining the quality of Australian and international students (Gatfield et al., 1999). It can be seen that “convergence management” has become one of the current directions of education management for studying abroad, but the receiving countries of international students must carry out personalized “convergence” from the existing educational development, cultural background and national traditions of their own countries.

### Cross-cultural adaptation of international students in China

The intercultural model constructed by Ward and colleagues (Searle and Ward, 1990; Ward and Kennedy, 1993; Masgoret and Ward, 2006) is recognized as one of the most influential concepts in intercultural adaptation. This model includes two dimensions, sociocultural adaptation and psychological adaptation. Aycan and Berry (1996) suggested adding a third dimension to this model, job adaptation (Aycan and Berry, 1996). Similarly, Black (1990) divided the sociocultural functions of foreigners in a new culture into general regulation (i.e., adaptation to general living conditions), interactive regulation (i.e., adaptation to social interactions with locals), and work regulation (Black, 1990). Based on the particularity of international students in China, this paper will understand the cross-cultural adaptation of international students from three aspects: psychological adaptation, cultural adaptation and learning adaptation.

The first is the psychological condition of international students in China. Researchers focus on the concept of “culture shock” and summarize subjective judgments on the phenomenon of “culture shock” for international students in China (Ward et al., 2020). On the other hand, researchers research the cross-cultural adaptation of international students in China based on the stress-coping framework (Peng and Wu, 2019). Western scholars pay more attention to the cross-cultural psychological adaptation of international students due to changes in the social and cultural environment. Geeraert et al. (2014) argue that the intercultural adaptation environment is a complex ecosystem, including family level, institutional level (work, school) and social level (ideology, policy, etc.; Geeraert et al., 2014). Culture shock can affect an individual’s stress adaptation in a new cultural environment (Ward et al., 2020). For example, international students are exposed to different cultures and are vulnerable to discrimination and prejudice, which is also one of the stressors (Bierwiaczonek and Waldzus, 2016). Specifically, Shafaei and Razak (2016) explained that lack of awareness of new cultural norms and values and differences between ethnic and new cultural norms and values could place international students in a challenging situation, resulting in Negative emotions and negative physiological responses (Shafaei and Razak, 2016).

The second is a cultural adaptation for international students in China. As an important part of the life of international students in China, social interaction has aroused the interest of scholars in its research (Wang and Lin, 2019). A language is an important tool for social interaction, yet most international students have little awareness or understanding of the importance of language and cultural strategies (Gong et al., 2021). They generally have limited access to cross-cultural mentoring, often focused on logistical aspects (e.g., transfer of credits, health and safety issues; Weissman et al., 2005; Bathurst and La Brack, 2012). Communicating with people with different linguistic and cultural backgrounds can lead to misattribution and hurt feelings due to limited social term awareness and intercultural competence (Jackson and Oguro, 2017). In addition, international students may develop “proficiency in self-expression and meeting their various social needs” in the host culture (Hanada and Horie, 2021), while continuing to experience a sense of boundary or “otherness” in the face of conflicting values and beliefs.” sense (Gu et al., 2010).

The third is for the study and life of international students in China. Conduct research on the adaptability of international students to China in terms of teaching methods, teaching language testing methods, and course content (Cargill et al., 2018; Huang et al., 2020). Foreign scholars mainly discussed the influencing factors, existing problems and targeted suggestions of cross-cultural communication (Ægisdóttir et al., 2008; Raymond and Hall, 2008; Parrish and Linder-VanBerschot, 2010; Matsumoto and Hwang, 2013; Ruiz et al., 2021). In teaching and learning practice, teachers usually use three methods to teach internationalized curriculum content: additional methods, curriculum installation methods and transformation methods (Macgregor and Folinazzo, 2018), and differences in cultural adaptation are not the only factors determining the quality of learning (Herdman et al., 1998).

### Research on educational management problems and countermeasures for international students in China

Scholars mainly focus on the management of international students from four perspectives, namely enrollment management, teaching management, and student management. Life management and employment management analyze the problems, causes, and countermeasures at various management levels.

First of all, in terms of enrollment management, Zhang Linlin (2018) believes that the quality of international students studying in China is uneven, the publicity during enrollment is weak, and there is an imbalance in enrollment majors (Zhang Linlin, 2018). For this reason, it is necessary to strengthen the investment of enrollment resources. Scholars such as Li and Xue (2021) and Li et al. (2022) divided China’s “double first-class” universities into four categories according to their admissions policies, namely “active and rigorous,” “actively rigorous” and “inactive,” and the discussion pointed out that China lacks national standards in recruiting international students (Li and Xue, 2021; Li et al., 2022). Ross et al. (2007) explored the institutional and managerial factors influencing international student admissions (Ross et al., 2007). Two scholars, Gao and Liu (2020), considering the huge capital brought by international students, suggested that universities in different countries adopt different strategies to attract international students and increase their market share, including creating a campus with an academic and social support atmosphere, participate in international education fairs and admissions events to expand the scale of international student admissions (Gao and Liu, 2020).

Secondly, in terms of teaching management, Zhu and Engels (2014) proposed strategies to strengthen the construction of international courses and innovate diversified teaching methods based on Kelly’s attribution theory, aiming at the problems existing in international students’ teaching practice in colleges universities (Zhu and Engels, 2014). Two scholars, Tran and Wall (2019) studied the educational philosophy of ubuntu, an African worldview, and regarded it as a key principle for the adoption of teaching practice in the teaching of international students, pointing out that this practice is beneficial to the teaching of international students (Tran and Wall, 2019).

Finally, in terms of life management, Sundquist et al. (2018) proposed that it is necessary to improve the system construction of logistics management, clarify the education management system, and actively guide international students to improve their quality and overcome maladaptation in education management (Sundquist et al., 2018). Such as establishing reasonable expectations, strengthening a learning, respecting cultural differences, seeking common ground while reserving differences, enhancing emotional exchanges, and reducing conflict between words and deeds. Ammigan and Jones (2018) pointed out that universities in the US, UK and Australia often have dedicated offices (Ammigan and Jones, 2018). These offices often provide a wide range of services, from advice on immigration compliance, academic, employment, financial and personal issues to hosting social and cultural programs to help students adjust to a different cultural environment as quickly as possible (Ammigan and Jones, 2018).

## Materials and methods

### Procedure

**Figure 1:**
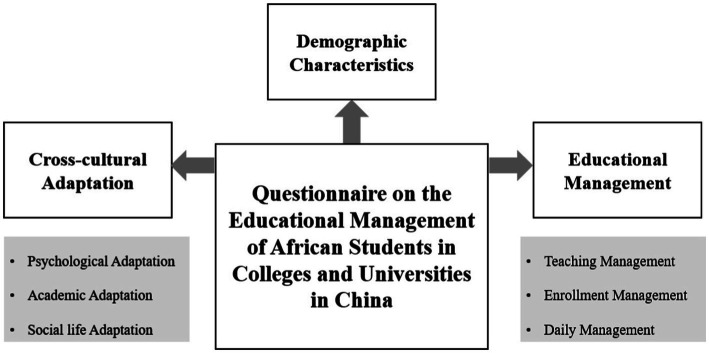
Education management of African student at Chinese University.

The questionnaires of this study were distributed through the Tencent questionnaire platform. The distribution period is from January 1, 2022, to February 15, 2022. We gathered a large sample because most of international students were in winter vacation. There was no reward mechanism or psychological suggestion to the participants during the questionnaire distribution process. The participants completed the survey in about 8 to 15 min. The Ethics Committee of the University’s College of Teacher Education approved the study, and it followed the Declaration of Helsinki. More details regarding the questionnaire on the educational management of African students in the Figure 1.

### Participants

In this study, participants from 13 Colleges of Zhejiang Normal University were selected as the survey objects, and a questionnaire survey was carried out. Zhejiang Normal University was the main data collection point and field research point. A total of 531 questionnaires were recovered, and after excluding invalid questionnaires, only 475 samples were validated. Specially, more than 90% of the valid samples are between 18 and 35 years old, and the number of male African international students in China exceeds that of women, 280 males (59%) and 195 females (41%) participated in this study. In terms of majors, social sciences, humanities and education are popular majors for them. In terms of education level, 100% of African students studying in China have a bachelor’s degree or above, which shows that international students generally have high learning literacy, and the group of international students has gradually shifted from elite to popular. About 86% of the African students stay in China for more than 1 year, which shows that most of them have a basic understanding and adaptation to China. In terms of language, more than half of the African students in China have reached the intermediate and advanced level of Chinese, but about 40% of them still have a low level of Chinese, which is likely to make difficulties to their lives and studies in China.

### Measurement

**Figure 2:**
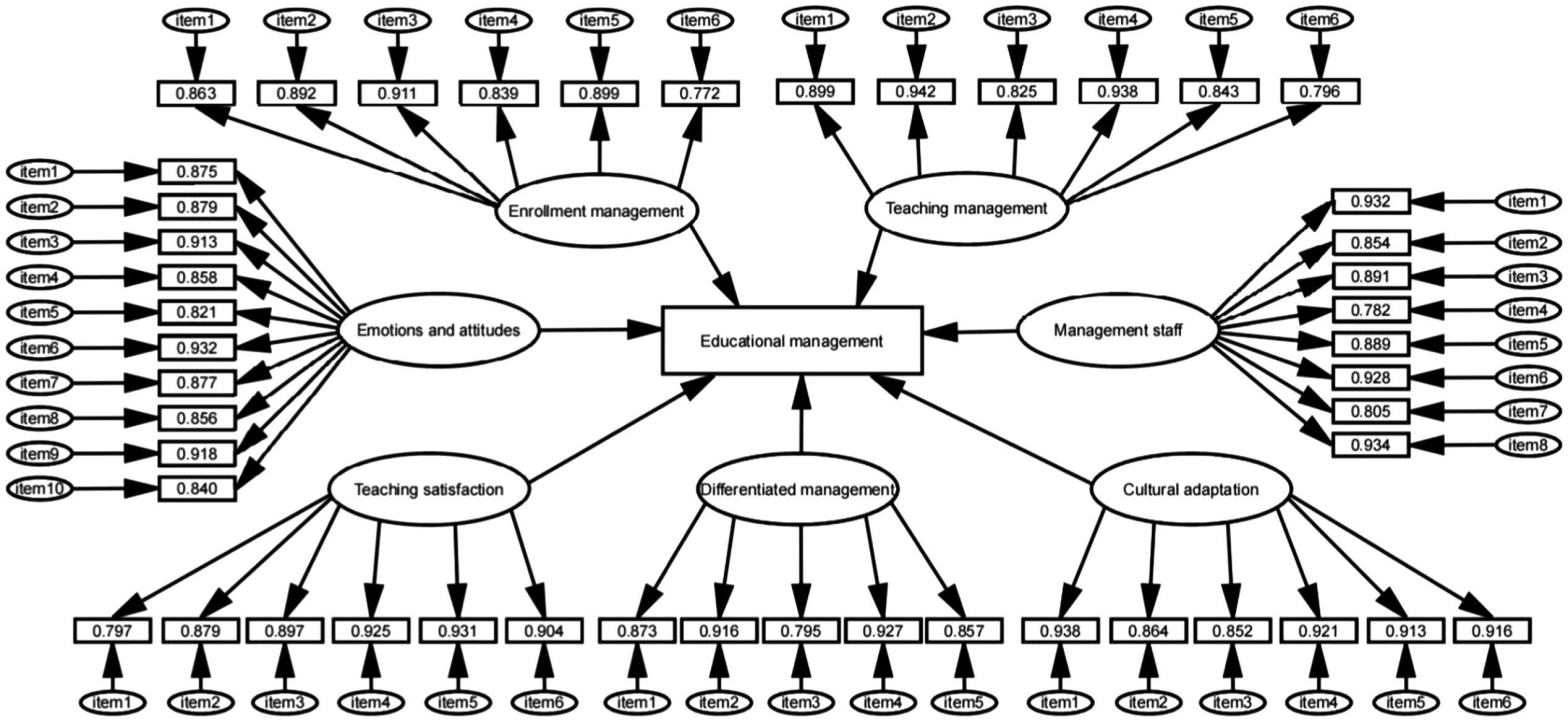
Educational management measurement tools.

Confirmatory factor analysis CFA was tested to confirm the validity of our measurement model, and also tested comparative fit index, Standardized root mean square error of approximation, Tucker-Lewis index, Goodness of fit index, Expected cross validation index and Root mean square error of approximation for Enrollment Management, Teaching Management, Differentiated Management, Management Staff, Teaching Satisfaction, Cultural Adaptation and Emotions and Attitudes. From the findings (Figure 2), the factor loading of each item is more than 0.7 and it represents the high degree of explanation for the observed variables.

Using factor loading and error variances to calculate estimated composite reliability (CR) and average variance extracted (AVE). The result of the estimated composite reliability value (CR) of each variable is more than 0.9, which achieves to a good level of internal consistency and shows that the model has good aggregation validity. The average variance extracted (AVE) of each variable is more than 0.7 and the square root of it is greater than the corresponding correlation coefficient of the variable, which indicating that the model has good discriminant validity. More details are shown in Table 1.

**Table 1:** Average variance extracted, composite reliability (*N* = 475).

Dimension	Item	Estimate	AVE	CR
EM	I can clearly understand the application requirements and application process of the university.	0.863	0.7464	0.9463
	I can solve the problem by visiting the registration website, email or telephone consultation.	0.892		
	I can have a clear and complete understanding of my professional training program.	0.911		
	I can clearly understand the employment status of my major from the school’s recruitment publicity.	0.839		
	I can clearly understand the scholarship Settings from the school’s recruitment promotion.	0.899		
	I can clearly understand the tutor information from the school’s recruitment promotion.	0.772		
TM	My tutor often takes the initiative to communicate with me.	0.899	0.7667	0.9515
	The textbook suits me to study.	0.942		
	The proportion of theory and practice in the classroom is reasonable.	0.825		
	I have the opportunity to comment on the faculty and the curriculum.	0.938		
	I have access to a wealth of scientific information.	0.843		
	I have ample opportunity to participate in scientific research.	0.796		
MS	The staff handled the business efficiently.	0.932	0.7945	0.9685
	The transaction process is simple.	0.954		
	The staff are friendly.	0.891		
	Staff can define job responsibilities.	0.782		
	The staff can communicate with me without difficulty.	0.889		
	All departments cooperate well.	0.928		
	Staff accept suggestions and improve them promptly.	0.805		
	The staff will not shirk their responsibilities.	0.934		
DM	I have participated in student organizations in China.	0.873	0.7654	0.9421
	I often participate in activities with Chinese students.	0.916		
	I share the canteen with Chinese students.	0.795		
	I live in the same dormitory with Chinese students.	0.927		
	I always take classes with Chinese students.	0.857		
CA	I can discuss with Chinese students.	0.938	0.8122	0.9628
	I can make friends with Chinese students.	0.864		
	I can shop smoothly.	0.852		
	I can use social software (QQ, wechat, etc.) smoothly.	0.921		
	I am getting to know Chinese culture.	0.913		
	I can adapt myself to the local cuisine.	0.916		
TS	I can hold on to class.	0.797	0.792	0.958
	I can stay focused in class.	0.879		
	I can finish my homework on time.	0.897		
	I can pass the exam.	0.925		
	I can understand the teaching content smoothly.	0.931		
	I am motivated to learn.	0.904		
EA	I always feel lonely.	0.875	0.7701	0.971
	I cannot talk about religion.	0.879		
	I feel tired for no reason.	0.913		
	I feel a lot of mental pressure.	0.858		
	I cannot keep my cool sometimes.	0.821		
	I get angry easily.	0.932		
	I’m hopeful about the future.	0.877		
	I feel like I’m indispensable.	0.856		
	I feel my life is meaningful.	0.918		
	I still like what I usually like.	0.84		

EM means Enrollment management, TM means Teaching management, MS means Management Staff Dealing with International Student Affairs, DM means Differentiated Management, CA means Cultural Adaptation, TS means Teaching Satisfaction, EA means Emotions and Attitudes.

#### Enrollment management

Enrollment management has a total of 6 topics, mainly to investigate the enrollment path and enrollment guidance of international students in colleges and universities. This dimension was designed using a Likert five-scale scale, in which respondents indicated their level of agreement or disagreement with each topic (1 = strongly agree, 5 = strongly disagree). The Cronbach’s alpha for this scale was 0.865, the CR = 0.9463 and the AVE = 0.7464.

The results of confirmatory factor analysis (CFA) showed that comparative fit index CFI = 0.95, standardized root mean square error of approximation (SRMR) = 0.03, Tucker-Lewis Index (TLI) = 0.91, goodness of fit index (GFI) = 0.92, Expected cross validation index (ECVI) = 0.82, and root mean square error of approximation (RMSEA) = 0.03.

#### Teaching management

There are 6 topics in teaching management, which mainly investigate the situation of curriculum setting and the reception of academic information of international students. This dimension was designed using a Likert five-scale scale, in which respondents indicated their level of agreement or disagreement with each topic (1 = strongly agree, 5 = strongly disagree). The Cronbach’s alpha for this scale was 0.831, the CR = 0.9515 and the AVE = 0.7667.

The results of confirmatory factor analysis (CFA) showed that comparative fit index CFI = 0.98, standardized root mean square error of approximation (SRMR) = 0.01, Tucker-Lewis Index (TLI) = 0.93, goodness of fit index (GFI) = 0.94, Expected cross validation index (ECVI) = 0.62, and root mean square error of approximation (RMSEA) = 0.01.

#### Differentiated management

There are 5 questions in differentiated management, mainly to investigate the daily management of Chinese and international students. This dimension was designed using a Likert five-scale scale, in which respondents indicated their level of agreement or disagreement with each topic (1 = strongly agree, 5 = strongly disagree). The Cronbach’s alpha for this scale is 0.770, the CR = 0.9421 and the AVE = 0.7654.

The results of confirmatory factor analysis (CFA) showed that comparative fit index (CFI) = 0.96, standardized root mean square error of approximation SRMR = 0.03, Tucker-Lewis Index (TLI) = 0.92, goodness of fit index (GFI) = 0.92, expected cross validation index (ECVI) = 0.74, and root mean square error of approximation (RMSEA) = 0.01.

#### Management staff dealing with international student affairs

There are 8 questions for management staffs, mainly to investigate the degree of responsibility for the daily work of management staffs and the satisfaction of international students. This dimension was designed using a Likert five-scale scale, in which respondents indicated their level of agreement or disagreement with each topic (1 = strongly agree, 5 = strongly disagree). The Cronbach’s alpha for this scale was 0.933, the CR = 0.9685 and the AVE = 0.7945.

The results of confirmatory factor analysis (CFA) showed that comparative fit index CFI = 0.98, standardized root mean square error of approximation (SRMR) = 0.01, Tucker-Lewis Index (TLI) = 0.96, goodness of fit index (GFI) = 0.96, expected cross validation index (ECVI) = 0.71, and root mean square error of approximation (RMSEA) = 0.01.

#### Teaching satisfaction

The teaching satisfaction has 6 questions, mainly to investigate international students’ classroom engagement status and learning situation. This dimension was designed using a Likert five-scale scale, in which respondents indicated their level of agreement or disagreement with each topic (1 = strongly agree, 5 = strongly disagree). The Cronbach’s alpha for this scale was 0.889, the CR = 0.958 and the AVE = 0.792.

The results of confirmatory factor analysis (CFA) showed that comparative fit index CFI = 0.94, standardized root mean square error of approximation (SRMR) = 0.04, Tucker-Lewis Index (TLI) = 0.91, goodness of fit index (GFI) = 0.92, expected cross validation index (ECVI) = 0.84, and root mean square error of approximation (RMSEA) = 0.01.

#### Cultural adaptation

There are 6 questions on cultural adaptation, mainly investigating the international students’ adaptability to life in China and their understanding of Chinese culture. This dimension was designed using a Likert five-scale scale, in which respondents indicated their level of agreement or disagreement with each topic (1 = strongly agree, 5 = strongly disagree). The Cronbach’s alpha for this scale was 0.861, the CR = 0.9628 and the AVE = 0.8122.

The results of confirmatory factor analysis (CFA) showed that comparative fit index CFI = 0.97, standardized root mean square error of approximation (SRMR) = 0.01, Tucker-Lewis Index (TLI) = 0.96, goodness of fit index (GFI) = 0.95, expected cross validation index (ECVI) = 0.75, and root mean square error of approximation (RMSEA) = 0.02.

#### Emotions and attitudes

There are 10 questions in emotional attitude, mainly to analyze international students’ psychological and emotional status and changes. This dimension was designed using a Likert five-scale scale, in which respondents indicated their level of agreement or disagreement with each topic (1 = strongly agree, 5 = strongly disagree). The Cronbach’s alpha for this scale was 0.785, the CR = 0.9710 and the AVE = 0.7701.

The results of confirmatory factor analysis (CFA) showed that comparative fit index CFI = 0.95, standardized root mean square error of approximation (SRMR) = 0.02, Tucker-Lewis Index (TLI) = 0.92, goodness of fit index (GFI) = 0.92, expected cross validation index (ECVI) = 0.80, and root mean square error of approximation (RMSEA) = 0.03.

### Data analysis

We used SPSS 26, JASP 0.16 and AMOS 24 for the statistical analysis of the data. The differences analysis of gender, major, and other demographic variables in the test variables of teaching satisfaction, cultural adaptation, emotions and attitudes was mainly carried out. Pearson Correlation analysis and Spearman Correlation analysis analyzed the Ordinal variables such as age, educational background, and time in China, Chinese proficiency, enrollment management, teaching management, differentiated management, management staffs, teaching satisfaction, cultural adaptation, emotions and attitudes and other indicators. The correlation between the Scalar variables was measured, and the multiple linear regression method was adopted to estimate the effect of each factor on the independent variable.

## Results

### Basic analysis of questionnaire data

#### Enrollment management

The overall mean score for enrollment management was 3.55 (SD = 0.82). The average score for men was 3.64 (SD = 0.89), and the average score for women was 3.41 (SD = 0.70). There was no significant difference between genders (*p* > 0.05). The average score for humanities majors is 3.88 (SD = 0.79), the average score for science is 3.67 (SD = 0.56), the average score for social sciences is 3.39 (SD = 0.81), the average score for engineering is 3.87 (SD = 0.93), and the average score for arts is 3.87 (SD = 0.93). The average score of sports was 3.52 (SD = 0.49), and the average score of others was 3.30 (SD = 0.86). There was no significant difference between different majors (*F* = 1.700, *p* = 0.142). There was a significant positive correlation between enrollment management recognition and time in China (*p* < 0.01), but no significant relationship with age, education, and Chinese proficiency. The correlation analysis is shown in Table 2.

**Table 2:** Means, standard deviations, and correlations (*N* = 475).

#	Variables	M	SD	1	2	3	4	5	6	7	8	9	10	11	12	13
1	G			1		-	-									
2	P				1											
3	Age	2.64	0.612			1										
4	EL	2.55	0.609				1									
5	T	3.22	0.949					1								
6	CP	3.10	1.586						1							
7	EM	3.54	0.825			0.106	0.295^**^	−0.025	0.135	1						
8	TM	3.36	0.813			0.246^*^	0.251^*^	−0.108	0.029	0.658^**^	1					
9	DM	3.02	0.993			−0.096	0.066	0.084	0.252^*^	0.435^**^	0.402^**^	1				
10	MS	3.31	0.994			0.007	0.091	0.033	0.169	0.667^**^	0.568^**^	0.474^**^	1			
11	TS	4.10	0.806			0.051	0.095	0.106	−0.026	0.479^**^	0.458^**^	0.216^**^	0.514^**^	1		
12	CA	3.71	0.927			0.026	0.140	0.127	0.357^*^	0.454^**^	0.430^**^	0.529^**^	0.545^**^	0.537^**^	1	
13	EA	3.60	0.728			0.225^*^	0.105	0.201^*^	0.011	0.177	0.365^**^	0.014	0.291^**^	0.351^**^	0.291^**^	1

M means Mean, SD means standard deviation, G means Gender, P means professional, EL means Educational level, T means Time spent in China, CP means Chinese proficiency, EM means Enrollment management, TM means Teaching management, DM means Differentiated Management, MS means Management Staff Dealing with International Student Affairs, TS means Teaching Satisfaction, CA means Cultural Adaptation, EA means Emotions and Attitudes.

* *p* < 0.05;

** *p* < 0.01.

#### Teaching management

The overall mean score for teaching management was 3.36 (SD = 0.80). The average score for men was 3.43 (SD = 0.89), and the average score for women was 3.24 (SD = 0.66), with no significant difference (*p* > 0.05). The average score for humanities majors is 3.76 (SD = 0.67), the average score for science is 3.21 (SD = 0.84), the average score for social sciences is 3.21 (SD = 0.83), the average score for engineering is 3.49 (SD = 0.52), and the average score for arts is 3.49 (SD = 0.52). The average score of sports was 3.27 (SD = 0.78), and the average score of others was 3.20 (SD = 0.96), and there was no significant difference (*F* = 1.512, *p* = 0.193). Teaching management recognition was significantly positively correlated with age and educational background (*p* < 0.05) and was significantly positively correlated with enrollment management recognition (*p* < 0.01) but had no significant relationship with time in China and Chinese proficiency. The correlation analysis is shown in Table 2.

#### Differentiated management

The overall mean score for differentiated management was 3.02 (SD = 0.99). The average score for men was 3.13 (SD = 1.01), and the average score for women was 2.85 (SD = 0.95), with no significant difference (*p* > 0.05). The average score for humanities majors is 3.34 (SD = 0.96), the average score for science is 2.80 (SD = 0.54), the average score for social sciences is 2.97 (SD = 1.01), the average score for engineering is 3.07 (SD = 0.60), and the average score for arts is 3.07 (SD = 0.60). The average score of sports was 2.78 (SD = 1.18), and the average score of others was 2.88 (SD = 1.19), and there was no significant difference (*F* = 0.641, *p* = 0.669). The recognition of differentiated management was significantly positively correlated with Chinese proficiency (*p* < 0.05) and was positively correlated with enrollment management and teaching management (*p* < 0.01) but had no significant relationship with age, education, and time in China. The correlation analysis is shown in Table 2.

#### Management staff dealing with international student affairs

The overall average score of management staffs was 3.31 (SD = 0.99), among which the average score of males was 3.40 (SD = 1.05), and the average score of females was 3.18 (SD = 0.91), with no significant difference (*p* > 0.05). The average score for humanities majors is 3.65 (SD = 0.94), the average score for science is 2.94 (SD = 0.85), the average score for social sciences is 3.25 (SD = 0.93), the average score for engineering is 3.47 (SD = 0.99), and the average score for arts is 3.47 (SD = 0.99). The average score of sports was 3.60 (SD = 0.91), and the average score of others was 2.91 (SD = 1.18), and there was no significant difference (*F* = 1.405, *p* = 0.230). The recognition of management staffs was significantly positively correlated with enrollment management, teaching management, and differentiated management (*p* < 0.01) but had no significant relationship with age, education, time in China, and Chinese proficiency. The correlation analysis is shown in Table 2.

#### Teaching satisfaction

The overall mean score for teaching satisfaction was 4.10 (SD = 0.81). The average score for men was 4.18 (SD = 0.81), and the average score for women was 3.98 (SD = 0.80), with no significant difference (*p* > 0.05). The average score for humanities majors is 4.34 (SD = 0.66), the average score for science is 4.17 (SD = 0.27), the average score for social sciences is 4.07 (SD = 0.73), the average score for engineering is 3.94 (SD = 1.14), and the average score for arts is 3.94 (SD = 1.14). The average score of sports was 3.98 (SD = 0.83), and the average score of others was 4.03 (SD = 0.97), and there was no significant difference (*F* = 0.513, *p* = 0.766). Teaching satisfaction was significantly positively correlated with enrollment management, teaching management, differentiated management, and recognition of management staffs (*p* < 0.01), but had no significant relationship with age, education, time in China, and Chinese proficiency. The correlation analysis is shown in Table 2.

#### Cultural adaptation

The overall mean score for cultural fitness was 3.71 (SD = 0.93). Among them, the average score of men was 3.82 (SD = 0.89), and the average score of women was 3.56 (SD = 0.97), and there was no significant difference (*p* > 0.05). The average score for humanities majors is 4.01 (SD = 0.65), the average score for science is 3.75 (SD = 0.96), the average score for social sciences is 3.68 (SD = 0.85), the average score for engineering is 3.64 (SD = 1.14), and the average score for arts is 3.64 (SD = 1.14). The average score of sports was 3.48 (SD = 1.21), and the average score of others was 3.61 (SD = 1.12), and there was no significant difference (*F* = 0.568, *p* = 0.724). Cultural adaptability was significantly positively correlated with Chinese proficiency (*p* < 0.05) and was significantly positively correlated with enrollment management, teaching management, differentiated management, recognition of management staffs, and teaching satisfaction (*p* < 0.01). There is no significant relationship between age, education and time in China. The correlation analysis is shown in Table 2.

#### Emotions and attitudes

The overall mean score for emotional attitude was 3.59 (SD = 0.73). The average score for men was 3.67 (SD = 0.94), and the average score for women was 3.47 (SD = 0.11), and there was no significant difference (*p* > 0.05). The average score for humanities majors is 3.72 (SD = 0.76), the average score for science is 3.40 (SD = 0.65), the average score for social sciences is 3.52 (SD = 0.77), the average score for engineering is 3.80 (SD = 0.63), and the average score for arts is 3.80 (SD = 0.63). The average score of sports was 3.30 (SD = 0.63), and the average score of others was 3.65 (SD = 0.72), and there was no significant difference (*F* = 0.699, *p* = 0.625). The emotional attitude was significantly correlated with age and time in China (*p* < 0.05), and was significantly positively correlated with teaching management, the recognition of management staffs, teaching satisfaction, and cultural adaptation (*p* < 0.01), and was significantly positively correlated with academic qualifications, there was no significant relationship between Chinese proficiency. The correlation analysis is shown in Table 2.

### Linear regression analysis

#### Teaching satisfaction

According to the results of the correlation analysis, we have made a simple stepwise linear regression analysis on the dependent variable teaching satisfaction and the independent variables of enrollment management, teaching management, differentiated management, and the recognition of management staffs. The fitting degree of this linear regression model is 0.332, there is no multicollinearity among the independent variables (VIF < 5), and the regression equation is significant (*p* < 0.01), which means that at least one of the independent variables can significantly affect the dependent variable. The recognition degree of enrollment management (β > 0, *p* < 0.05) and the recognition degree of management staffs (β > 0, p < 0.05) can positively affect teaching satisfaction. The linear regression analysis is shown in Table 3. The regression equation is:

**Table 3:** Testing the effects of the predictors in the model on Teaching satisfaction.

Effect	UC		SC	t	Sig.	CS	
	Beta	SE	Beta			Tolerance	VIF
(Constant)	2.119	0.301		7.040	<0.01		
EM	0.346	0.110	0.350	3.149	0.002	0.558	1.792
MS	0.228	0.090	0.282	2.536	0.013	0.558	1.792
R^2^				0.332			
F				24.157			
P				<0.01			
Dependent Variable: TS							

UC means Unstandardized Coefficients, SC means Standardized Coefficients, CS means Collinearity Statistics, EM means Enrollment management, MS means Management Staff Dealing with International Student Affairs, TS means Teaching satisfaction.

Teaching Satisfaction = 2.119 + 0.346*Enrollment Management Recognition + 0.228*International Student Affairs Manager Recognition.

#### Cultural adaptation

According to the results of the correlation analysis, we made a simple stepwise linear regression analysis on the dependent variable cultural fitness, the control variable Chinese proficiency, the independent variables enrollment management, teaching management, differentiated management, and the recognition of international students’ affairs managers. The degree of fit of the regression model was 0.444, there was no multicollinearity among the independent variables (VIF < 5), and the regression equation was significant (*p* < 0.01), meaning that at least one of the independent variables could significantly affect the dependent variable. Chinese proficiency (β > 0, *p* < 0.05), recognition of management staffs (β > 0, *p* < 0.05), and recognition of differentiated management (β > 0, *p* < 0.05) can positively affect teaching satisfaction. The linear regression analysis is shown in Table 4. The regression equation is: Cultural adaptability = 1.346 + Chinese proficiency * 0.140 + recognition of differentiated management * 0.268 + recognition of management staffs * 0.340.

**Table 4:** Testing the effects of the predictors in the model on Cultural adaptation.

Effect	UC		SC	t	Sig.	CS	
	Beta	SE	Beta			Tolerance	VIF
(Constant)	1.346	0.280		4.802	<0.01		
CP	0.140	0.047	0.240	3.010	0.003	0.913	1.095
DM	0.268	0.083	0.287	3.235	0.002	0.734	1.363
MS	0.340	0.081	0.364	4.209	<0.01	0.772	1.295
R^2^				0.444			
F				25.601			
P				<0.01			
Dependent Variable: CA							

UC means Unstandardized Coefficients, SC means Standardized Coefficients, CS means Collinearity Statistics, CP means Chinese proficiency, DM means Differentiated Management, MS means Management Staff Dealing with International Student Affairs, CA means Cultural adaptation.

#### Emotions and attitudes

According to the results of the correlation analysis, we made a simple step-by-step linear regression analysis on the emotions and attitudes of the dependent variables, the control variables of age and time in China, the independent variables of enrollment management, teaching management, differentiated management, and the recognition of management staffs. The degree of fit of the sublinear regression model was 0.122, there was no multicollinearity among the independent variables (VIF < 5), and the regression equation was significant (*p* < 0.01), meaning that at least one of the independent variables could significantly affect the dependent variable. Teaching management recognition (β > 0, *p* < 0.05) can positively affect emotionas and attitudes. The linear regression analysis is shown in Table 5. The regression equation is: Emotions and attitudes = 2.533 + teaching management recognition * 0.314.

**Table 5:** Testing the effects of the predictors in the model on Emotions and attitudes.

Effect	UC		SC	t	Sig.	CS	
	Beta	SE	Beta			Tolerance	VIF
(Constant)	2.533	0.294		8.608	<0.01		
TM	0.314	0.085	0.349	3.692	<0.01	1.000	1.000
R^2^				0.122			
F				13.629			
P				<0.01			
Dependent Variable: EA							

UC means Unstandardized Coefficients, SC means Standardized Coefficients, CS means Collinearity Statistics, TM means Teaching management, EA means Emotions and attitudes.

## Discussion

### Problems in enrollment management

Comparing Crowley (2021) in his study, the enrolment management in private mom-profit higher education, a Lack of enrollment channels in Zhejiang Normal University’s African students mainly obtain relevant information about studying abroad by browsing the school’s website and submitting their study abroad application. In fact, there are various channels for African international students to apply for universities and colleges in China, such as applying on the school website, consulting educational institutions, participating in exchange programs, winning scholarships, listening to expert advice, and being recommended by classmates or alumni. However, compared with the friendly exchanges and inter-school exchanges of African international students in other colleges and universities in China or other countries, Zhejiang Normal University has fewer exchange channels to enroll international students. In addition, the effect of international scholarship in the recruitment of international students has not reached the expected state, it does not play a good role in attracting students to study abroad. Harvey-Smith (2022) also discussed the reimagining strategic enrollment management in colleges and universities and compared with our study how to enrich enrollment channels and keep international students optimistic about the success of their applications is also an important issue that colleges and universities generally face when recruiting African students to study in China.

Wen and Dai (2021) discussed the innovative practice and promotion countermeasures of micro-major in Chinese universities and the importance of social media in enrolment promotion, although most African international students in China said that they can clearly understand the admission requirements and application process of universities and can also consult relevant questions to the schools they are applying for by phone, email, etc., they are not aware of the training plans and employment conditions of the majors they are applying to study. The lack of understanding means that Zhejiang Normal University has not made sufficient preparations for enrollment promotion, and there are certain deficiencies in the publicity work. Since Zhejiang Normal University rarely organizes delegations to African countries for educational promotion or educational exhibitions, it still does not have enough cooperation and exchanges with universities in African countries, it only carries out publicity through Chinese embassies and consulates in Africa and the introduction of African alumni. With the continuous development of the information age, these approaches have been far from being able to fully meet the needs of enrollment. Enrollment publicity is an indispensable way to raise awareness of colleges and universities and attract foreign students. Therefore, more attention should be paid to enroll promotion. And starting from the enrollment process, colleges and universities should maintain a responsible attitude towards international students and enrich the enrollment publicity materials on relevant platforms so as to provide convenience for African students to fully understand the actual situation of the universities and majors they apply for.

Following deficiencies are concretely reflected in Students’ teaching satisfaction (Table 4). Whether or not to have a good understanding in major for students is often related to their prior knowledge of the course, and a clear awareness of specialized training plan and tutor information helps them to plan their studying accordingly and define the direction and goals of research. Expanding the channels of information regarding enrollment, studying exchanges and international scholarship, and refining major introductions to ensure that African international students choose their majors on the basis of comprehensive understandings of major training programs and employment conditions. It’s helpful for optimizing enrollment management, cultivating students’ confidence in learning, and improving teaching satisfaction.

### Problems in teaching management

Rahmatullah et al. (2022) discussed the contribution of education and student psychology and as shown in Table 5, there is a significant connection between teaching management and students’ psychology. Unrealistic teaching schedule, a dearth of tutorial and language barrier are likely to cause feelings of pessimism and anxiety, which makes teaching management more difficult. The unreasonable curriculum setting is one of the factors affecting teaching management. On the one hand, practical teaching needs to be strengthened. At present, Chinese universities, including Zhejiang Normal University, organize African students to conduct professional internships and participate in educational practice infrequently. Practice is the best way to master and test knowledge. For African students, they are eager to get the chance to practice, especially for those African students who want to seek employment opportunities in China, internship is the best opportunity for them to practice. However, whether it is a liberal art major or a science major (physics major, chemistry major, mathematics major), the teaching of theoretical knowledge accounts for the main body of the class, and it is more common to follow the script. Such a teaching method will make the classroom boring and students’ learning efficiency low. And African students are more likely to have a rebellious mentality and violate the classroom teaching requirements. Therefore, flexible and targeted practice teaching is needed urgently.

On the other hand, the difficulty and fit of the course need to be further improved. African students studying in China with the same major have different study and living backgrounds and different original majors and educational levels. However, most domestic colleges and universities adopt undifferentiated class teaching methods and uniformly stipulate the teaching content, and the curriculum set still cannot get rid of the original subject system and meet the needs of individual development of African students. The differences in political and economic systems between Chinese and African countries are also reflected in the setting of disciplines and majors, which results in a disconnect between the content of courses taught by African students in their home countries and those taught in China. It be leads to a risky condition that African students cannot keep up with the current teaching progress, falling into a low spirit. In addition, colleges and universities have neglected to cultivate the scientific research ability of African students. The cultivation of scientific research ability should become an important part of the current professional study of international students, which can stimulate the learning initiative, accelerate the combination of theory and practice, cultivate innovative consciousness, develop innovative thinking, and promote the all-round development of students. However, due to the lack of support and channels, and the fact that universities do not regard the cultivation of scientific research ability as a teaching goal, it has led to the lack of scientific research ability of international students in colleges and universities to a certain extent. Colleges and universities should attach importance to cultivating African students’ practical and scientific research capabilities and choose teaching content based on their actual needs.

Chu (2022) cited that applying positive psychology to foster student’s engagement, the tutors are responsible for the inadequate implementation of the tutorial system. First, the comprehensiveness and depth of the tutor’s guidance to international students are not enough. As the leader of international students’ learning journey, college tutors should not only simply teach professional knowledge but also care about the learning methods, learning behaviors, and learning goals of international students. Nevertheless, the relationship between some tutors and international students is not close enough. Apart from the interactions in the classroom, there is little contact in daily life, and the tutors lack understanding and care for the international students. Second, the tutors do not know enough about the personality characteristics of international students, resulting in a lack of targeted guidance. International students are different from domestic students in terms of study habits, way of thinking and knowledge level. This requires tutors to teach students according to their aptitude and give appropriate guidance according to their different learning interests and characteristics, and the date analysis has demonstrated that students who have more communications with tutors and receive more assistance and guidance from them are more optimistic towards study and life.

Hou (2022) mentioned the importance of democracy and international student for excellent teaching, and from our study the lack of teaching democracy: African students yearn for a classroom environment with free communication, and they want to be able to discuss any issues of interest to them on an equal footing with teachers in the classroom. However, affected by cultural differences and language barriers, most Chinese teachers adapt the teaching method to instil knowledge in African students directly and rarely listen to the opinions of international students. Lack of equal communication between teachers and students and failure to create a democratic teaching atmosphere make the actual teaching effect far from expectations. For the group of international students, teachers should improve teaching methods, allow international students to express their opinions fully, and create a democratic and pleasant classroom atmosphere, so that they can keep a good mood and learn in joy.

### Problems in the daily management

As mentioned by Sidhu and Gage (2021) Motivating faculty to adopt teaching innovation is important and the logistical support take an important part of the daily management, the logistical support has a direct effect on teaching satisfaction and cross-culture adaption for African students (Tables 2, 3) represented by three main ways. Firstly, lack of standardized management system and professional management personnel, at present, the international student management structure of Zhejiang Normal University consists of several different organizations and departments. The International Center and the College of Education are responsible for the daily management of international students, while the Graduate School, the College of International Education and the Academic Affairs Office are responsible for the teaching of international students. Such multi-subject management and organizational structure are too complicated for international students, and there will also be problems such as shirk and unclear responsibilities. In addition, the professional level of the management team is not high. The current international student management staffs are not full-time staff and have not received professional training before taking up their posts, so they do not have professional management philosophy and skills. They also have troubles in communication with international students, and it is difficult to provide services that satisfy international students. In addition, management staffs lack professional quality, and it is difficult to maintain a good service attitude when facing international students, which results a dilemma that students are unfamiliar with regular policies such as apartment regulations but usually feel no one to turn to, gradually become tired of life abroad. Therefore, it is urgent to establish a standardized management system for international students and train professional management personnel.

Secondly, the differences between Chinese and African cultures are not emphasized. Adaptation to daily life is the first difficulty and challenge African students face when coming to China. From the survey results, international students have difficulties adapting to the local climate, transportation, shopping, accommodation, social activities, and pace of life. There are significant differences in the daily life of Chinese and African people. Therefore, African students who come to China to study in China will inevitably experience many difficulties. Huge cultural differences can also lead to mental health problems like fear of talking to fellows for international students. In daily management, the management department should pay attention to the cultural adaptation of international students, and provide them with convenience in terms of clothing, food, housing and transportation, such as improving the international service level of the canteen and school hospital, so as to help them adapt to the new life as soon as possible.

Thirdly, excessive differentiation management: As a special group of international students, colleges and universities should take appropriate measures to conduct differentiated management. However, due to inappropriate daily management systems and wrong management philosophy, Chinese students and international students are gradually isolated, making them two independent groups that do not interfere. For example, the establishment of independent teaching classes and independent student organizations makes it rare for African students to integrate into the social activities of native Chinese students. And the daily management system introduced only for international students has deepened the gap between Chinese students and African international students. Such kind of differentiated management has gradually become unfair and discriminatory, increases psychological stress, and even creates barriers for them to adapt to the local environment. Only humane and moderate differentiated management can convince international students.

## Conclusion

Under the background of “a community with a shared future for mankind,” international students in China, as pioneers and practitioners of human society’s cross-cultural communication, will surely participate more and more actively in global governance. Meanwhile, with the deepening of China-Africa friendly relations, more and more African students choose to study in China. Therefore, it is of great significance and value to study the education management of African students in colleges and universities. In this paper, the current research has yielded encouraging empirical results, which show that the teaching satisfaction of African students in China is closely related to enrollment management, teaching management, differentiated management, management staffs, and cultural adaptation. Furthermore, emotions and attitudes are correlated. At the same time, we found that the age, time and Chinese proficiency of African students studying in China are also important influencing factors, but the correlation is not as large as the previous factors. Our findings contribute to related research fields by illustrating that colleges and universities can further formulate and improve the educational management system for African students studying in China that is compatible with the development of higher education in China, create a systematic new form of educational management, and promote the internalization of the value of “a community with a shared future for mankind” to solve the current educational management problems of African students studying in China from the perspective of humanistic theory.

### Implication of the study

For the implication of this study from both theoretical and practical have a great significance to the psychological and educational management of international students in colleges and universities in China and other parts of the world. At present, with the increasing number of international students studying at Chinese universities and the expansion of the overall scale, the structure of international students and the training objectives of international students will inevitably change accordingly. However, the country’s theories on the education and management of international students in China are not perfect enough, and there is a lack of research on the depth and breadth of relevant theories. The actual development speed of international students’ education and management far exceeds the pace of theoretical research in China, and there is a lag in theoretical research. If the practice is guided by the educational management theories that are not suitable for reality, the effect will be counterproductive. In the management of education for African students, China should not only continue to expand and enrich the service content, but also pay more attention to the quality of service and constantly improve the level of educational management. Only in this way can China’s higher education greatly consolidate its international reputation and improve its international competitiveness. Otherwise, it is impossible to achieve long-term development if it is limited to the extension of scale and ignores the improvement of education quality and management norms.

This study incorporates the analysis of the educational management model of African students in China into the framework of “a community of shared future for mankind,” further formulates and improves the educational management system for African students in China that is compatible with the development of higher education in China. Specially, in enrollment management, colleges and universities should formulate scientific enrollment standards, improve the quality of enrollment services, widely use publicity media, and comprehensively expand enrollment and publicity channels to attract high-quality foreign talents to study in China. In teaching management, we should advocate the value of “harmony but difference,” construct diversified teaching models, and enhance teachers’ international understanding ability while cultivating their international vision from both psychological and educational practice. In daily management, relevant management personnel should adhere to the “people-oriented” service tenet, improve their self-quality through professional training, and actively build a platform for Chinese and foreign students to communicate. At the same time, colleges and universities should strengthen the construction of humanistic psychological care, eliminate the psychological barriers of African students studying in China, and make use of the excellent traditional Chinese culture to arouse the cultural resonance of African students, so as to promote students’ cross-cultural adaptation.

This research also conforms to the trend of the times and the development trend of the world, meets the needs of the society, and conducts a theoretical analysis on the implementation of exchanges and mutual learning in the process of China’s internationalization of higher education and the development of educational cooperation between China and Africa. The innovation and improvement of the university will promote the construction of a world of a long-term peace and stability and the common development of all countries in the world, promote the globalization of college education, and jointly build a community with a shared future for mankind.

### Limitations of the study

Although the research has sorted out and analysed the current situation and problems of the education, teaching and management mode of African international students in China from the perspective of empirical research, although it has a more comprehensive sampling range and larger sample size than previous research, it is affected by the information. Due to limited channels, the number of samples is still insufficient to a certain extent. Naturally, the data analysis results are still not accurate enough, which weakens the research conclusions’ reliability and the proposed strategies’ scientificity to a certain extent. What’s more, the questionnaire in this paper is based on a sample of one Chinese university, that is, it is only formulated for African international students from Zhejiang Normal University, which lacks representativeness. If more Chinese universities can be included and more samples can be collected, it is believed that more accurate conclusions can be obtained from the analysis results of relevant big data in the field.

## Data availability statement

The raw data supporting the conclusions of this article will be made available by the authors, without undue reservation.

## Ethics statement

The studies involving human participants were reviewed and approved by the Ethics Committee of the University’s College of Teacher Education. The studies followed the Declaration of Helsinki. The patients/participants provided their written informed consent to participate in this study.

## Author contributions

DW: writing-original draft preparation, conceptualization, formal analysis, methodology, funding acquisition, investigation and validation. ZS: writing-original draft preparation, conceptualization, methodology, review and editing. LX: investigation and data curation. OM: formal analysis, methodology, data analysis, review and editing, writing-original draft preparation. LD: writing-original draft preparation and investigation. DL: editing and integration. ZZ: data analysis and validation. YT: conceptualization, project administration, data curation and resources. All authors have read and agreed to the published version of the manuscript.

## Funding

This research was supported by Research on the Education and Management of African Students in China from the Perspective of Community of Shared Future for Mankind (19GXSZ11YB), a special project of Ideological and Political Work in Universities in Zhejiang Province Philosophy and Social Science Plan in 2019, and the Collaborative Innovation Center for African Studies and China-Africa Cooperation “Research on the Management countermeasures of Zhejiang International Students in the Normalization of the Epidemic” (21XTFZ05A).

## Conflict of interest

The authors declare that the research was conducted in the absence of any commercial or financial relationships that could be construed as a potential conflict of interest.

## Publisher’s note

All claims expressed in this article are solely those of the authors and do not necessarily represent those of their affiliated organizations, or those of the publisher, the editors and the reviewers. Any product that may be evaluated in this article, or claim that may be made by its manufacturer, is not guaranteed or endorsed by the publisher.
